# A deep learning strategy for accurate identification of purebred and hybrid pigs across SNP chips

**DOI:** 10.1186/s40104-025-01249-y

**Published:** 2025-08-14

**Authors:** Zipeng Zhang, Zhengwen Fang, Yongwang Du, Yilin He, Changsong Qian, Weijian Ye, Ning Zhang, Jianan Zhang, Xiangdong Ding

**Affiliations:** 1 State Key Laboratory of Animal Biotech Breeding, Key Laboratory of Animal Genetics and Breeding of Ministry of Agriculture and Rural Affairs, National Engineering Laboratory of Animal Breeding, College of Animal Science and Technology, China Agricultural University, Beijing, 100193 China; 2 MolBreeding Biotechnology Ltd., Shijiazhuang, 050035 China; 3 Henan Pig Biological Breeding Research Institute, Henan University of Animal Husbandry and Economy, Zhengzhou, 450046 China

**Keywords:** Breed identification, Genomic breed composition, Hybrid, Machine learning, Multi-output regression

## Abstract

**Background:**

Breed identification plays an important role in conserving indigenous breeds, managing genetic resources, and developing effective breeding strategies. However, researches on breed identification in livestock mainly focused on purebreds, and they yielded lower predict accuracy in hybrid. In this study, we presented a Multi-Layer Perceptron (MLP) model with multi-output regression framework specifically designed for genomic breed composition prediction of purebred and hybrid in pigs.

**Results:**

We utilized a total of 8,199 pigs from breeding farms in eight provinces in China, comprising Yorkshire, Landrace, Duroc and hybrids of Yorkshire × Landrace. All the animals were genotyped with 1K, 50K and 100K SNP chips. Comparing with random forest (RF), support vector regression (SVR) and Admixture, our results from five replicates of fivefold cross validation demonstrated that MLP achieved a breed identification accuracy of 100% for both hybrid and purebreds in 50K and 100K SNP chips, SVR performed comparable with MLP, they both outperformed RF and Admixture. In the independent testing, MLP yielded accuracy of 100% for all three pure breeds and hybrid across all SNP chips and panel, while SVR yielded 0.026%–0.121% lower accuracy than MLP. Compared with classification-based framework, the new strategy of multi-output regression framework in this study was helpful to improve the predict accuracy. MLP, RF and SVR, achieved consistent improvements across all six SNP chips/panel, especially in hybrid identification. Our results showed the determination threshold for purebred had different effects, SVR, RF and Admixture were very sensitive to threshold values, their optimal threshold fluctuated in different scenarios, while MLP kept optimal threshold 0.75 in all cases. The threshold of 0.65–0.75 is ideal for accurate breed identification. Among different density of SNP chips, the 1K SNP chip was most cost-effective as yielding 100% accuracy with enlarging training set. Hybrid individuals in the training set were useful for both purebred and hybrid identification.

**Conclusions:**

Our new MLP strategy demonstrated its high accuracy and robust applicability across low-, medium-, and high-density SNP chips. Multi-output regression framework could universally enhance prediction accuracy for ML methods. Our new strategy is also helpful for breed identification in other livestock.

**Supplementary Information:**

The online version contains supplementary material available at 10.1186/s40104-025-01249-y.

## Introduction

Accurate breed identification plays a pivotal role in biodiversity conservation of indigenous breeds [1, 2], systematic management of genetic resources [3], and development of evidence-based breeding programs [4]. The fundamental challenge in this field lies in characterizing inter-breed genetic heterogeneity. Conventional methodologies predominantly relied on phenotypic characterization, e.g. coat pigmentation and morphometric parameters. However, this approach was highly subjective and demonstrated limited efficacy for differentiating phenotypically similar breeds. Furthermore, it lacked the capacity to determine individual genomic ancestry composition. With the development of molecular markers, microsatellite markers emerged as a predominant tool for breed discrimination [5–8]. The recent paradigm shift towards high-throughput single nucleotide polymorphisms (SNPs) has revolutionized breed identification due to their cost-effectiveness and high genomic coverage [9–11]. Pioneering analytical approaches including linear regression models and admixture analysis were conventionally employed for genomic ancestry prediction [9, 12–15]. The regression-based method estimates genomic breed composition (GBC) through multivariate regression analysis of individual genotypes against reference population allele frequencies [16]. Admixture employs maximum likelihood estimation to infer ancestral proportions from genotypic data [13]. Nevertheless, significant limitations persist in current methodologies. Reverter et al. [14] reported that the linear regression method achieved a minimum misclassification rate of 3.704% for breed classification, while Kueh et al. [13] demonstrated that the maximum congruence between predicted and actual ancestry compositions using both approaches reached merely 92%.

The recent advancements in artificial intelligence (AI) have catalyzed widespread adoption of machine learning (ML) techniques, particularly for their superior capacity in handling high-dimensional genomic datasets and resolving complex nonlinear relationships. This methodological synergy has revolutionized animal breeding research, manifested in two principal applications: enhancing genomic selection accuracy [17, 18] and pinpointing candidate gene governing economically important traits [19, 20]. In the context of SNP-based breed identification, ML-based approaches have been increasingly implemented [11, 21–23], e.g., random forest (RF), support vector machines (SVM), k-nearest neighbors, partial least squares discriminant analysis (PLS-DA) and artificial neural network (ANN). Empirical evidence demonstrates the diagnostic potential of these methods, Wilmot et al. [24] reported 98.22% classification accuracy using PLS-DA in cattle breed discrimination. Comparable efficacy was observed in caprine studies, where ANN achieved 96.6% breed identification accuracy [25]. A comprehensive comparative analysis of six ML algorithms in pig breed classification revealed RF and SVM exhibited superior predictive performance and algorithmic robustness [26]. Nevertheless, existing methodologies demonstrate low accuracy in hybrid individual identification, exemplified by RF's 13.76% misclassification rate and complete classification failure for certain specimens [26]. This technical constraint stems from the enhanced genomic similarity between crossbreds and their parental purebred lines compared to distinct pure breeds. In addition, current approaches fail to meet the stringent requirement of 100% classification accuracy mandated by commercial livestock operations. These limitations highlight the critical need for novel computational frameworks capable of 100% accuracy in both purebred and hybrid classification.

In this study, we presented an innovative strategy for precise breed identification, employing a multi-output regression MLP model with three hidden layers specifically designed for GBC prediction. A comprehensive comparative analysis was conducted against prevailing ML-based classification approaches. Furthermore, we systematically evaluated the critical influence of purity determination thresholds on classification accuracy. Our investigation also extended to three key factors: 1) SNP marker density, 2) reference population size, and 3) ancestral diversity within reference cohorts, all of which significantly affect breed classification precision.

## Materials and methods

### Animals and genotypes

**Fig. 1:**
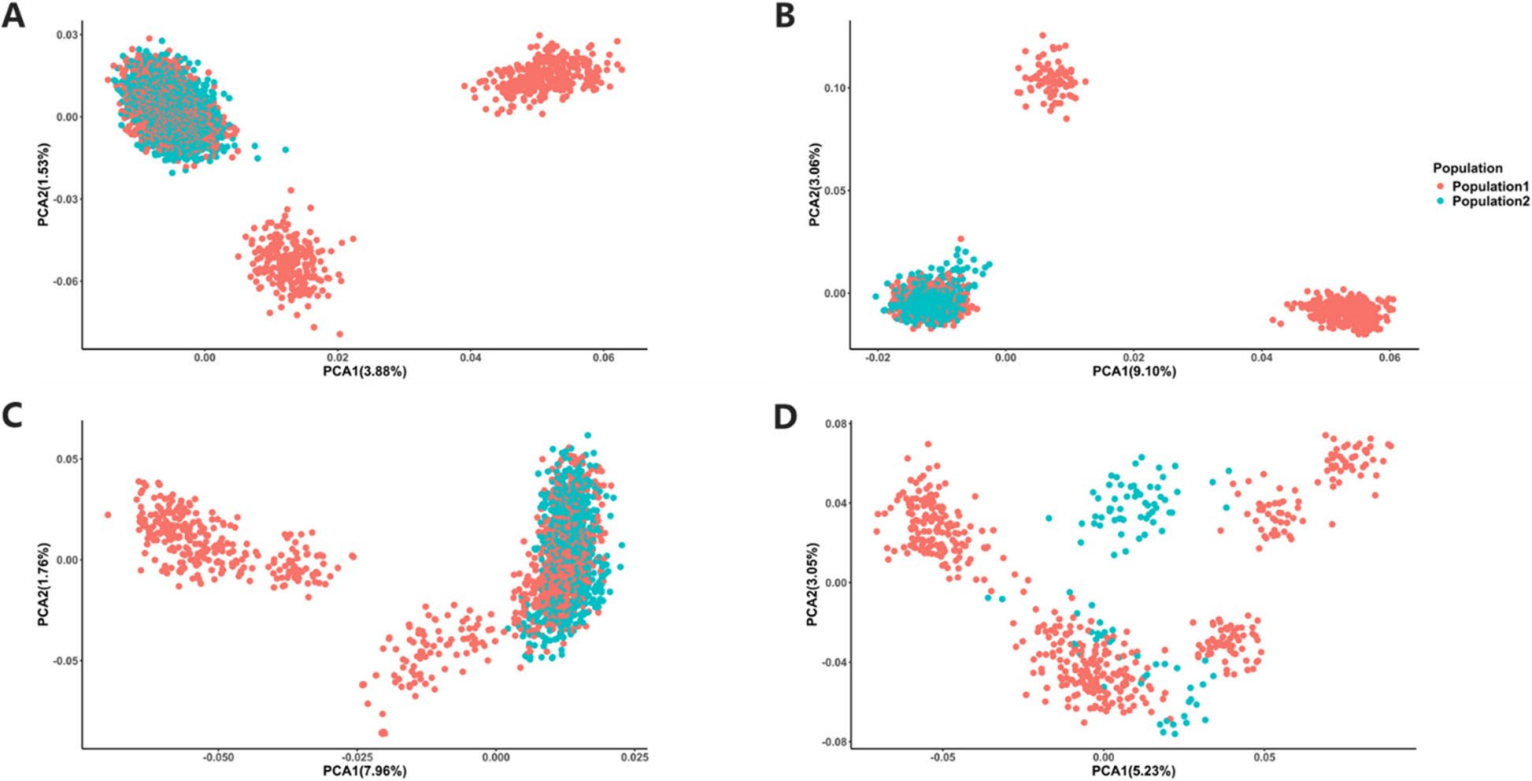
PCA for each breed in Population 1 and Population 2. **A** Yorkshire. **B** Landrace. **C** Duroc. **D** Hybrid

In this study, a total of 8,199 pigs sourced from breeding farms in eight provinces in China (Anhui, Hebei, Henan, Hunan, Guangdong, Jiangxi, Sichuan and Xinjiang), comprising 4,303 Yorkshire, 1,654 Landrace, 1,649 Duroc, and 593 hybrid pigs (Yorkshire × Landrace), were used. All individuals were genotyped using three 50K SNP panels, including Illumina Zhongxin No.1 50K (ZX50K), Illumina Porcine GGP 50K (GGP50K), Molbreeding GenoBaits^®^ Porcine DK 50K (GenoBaits50K) [27], in addition, all animals were genotyped using Molbreeding GenoBaits^®^ Porcine DK 1K (GenoBaits1K), and Molbreeding GenoBaits^®^ Porcine 100K (GenoBaits100K). The common SNPs among three 50K SNP chips were also used, referred as 50K_Common, including 18,422 SNPs. Genotype quality control was carried out using PLINK software [28]. SNPs with minor allele frequency less than 0.05, call rate less than 0.90, deviating from Hardy–Weinberg equilibrium (*P* < 10^–6^) were excluded and the deletion sites were imputed with NA. After genotype quality control 1,337 (GenoBaits1K), 50,287 (ZX50K), 45,743 (GGP50K), 50,962 (GenoBaits50K), 84,889 (GenoBaits100K) and 17,734 (50K_Common) SNPs remained on the above six different density SNP panels, respectively. Based on their origin, the pigs were divided into two populations: Population 1 and Population 2. Population 1 consisted of 2,185 Yorkshire, 1,111 Durocs, 1,102 Landraces, and 491 hybrids, in total 4,889 individuals. The left 3,310 individuals including 2,118 Yorkshires, 552 Landraces, 538 Durocs, and 102 hybrids were in Population 2. The PCA result indicated Populations 1 and 2 were genetic connected in all three pure breeds and hybrid (Fig. 1).

### Breed identification method

In this study, Admixture (v1.3.0) [13] and three ML methods including RF, Support Vector Regression (SVR), and our proposed Multi-Layer Perceptron (MLP) with three hidden layers were used to predict individual GBC. Individuals with a single breed ancestry greater than assigned threshold value for purebred were determined as purebreds, while those with lower threshold value were classified as hybrid.

Different from traditional classification-based framework, in which a single-label target variable is typically employed to represent a specific breed (e.g., Duroc, Landrace, Yorkshire, or hybrid) using one-hot encoding (e.g., [1,0,0,0]). In contrast, for all ML methods implemented in this study, we proposed the multi-output regression framework, which innovatively restructures the target variable by introducing multiple labels combined into a single vector. Each element in the vector corresponds to a purebred, precisely quantifying the GBC of a sample. Crucially, to ensure biological plausibility under Mendelian inheritance laws, the sum of all elements in the vector is constrained to 1. For instance, a binary hybrid individual composed of 50% Yorkshire and 50% Landrace would be encoded as [0.50, 0.50, 0.0]. Furthermore, unlike traditional classification models that rely on cross-entropy loss to maximize classification accuracy, our multi-output regression framework adopts the mean squared error (MSE) as the loss function, aiming to minimize the deviation between predicted ancestry proportions and actual ancestry proportions.

### Admixture

Admixture is a model-based clustering method that estimates the proportion of genetic ancestry from K hypothetical ancestral populations for each individual. In this research, K was set to 3, and the training set was used to determine the breeds represented by these ancestral populations.

### Random forest

RF uses voting or the average of multiple decision trees to determine the classification or predicted values of new instances [29]. It is essentially an ensemble of multiple decision trees, each trained with slightly different parameters. RF reduced the risk of overfitting by averaging the prediction results of many decision trees [30]. The model formulation of RF can be expressed as:

$$\boldsymbol y=\frac1M\sum\nolimits_{m=1}^M\;t_m(\psi_m(\boldsymbol y:\boldsymbol X))$$

where ${\varvec{y}}$ is the vector of breed composition, $t_m(\psi_m(\boldsymbol y:\boldsymbol X))$ is an individual decision tree, and $M$ is the number of decision trees in RF.

### Support vector regression

SVR is the application of support vector machine in regression to deal with quantitative responses, using a linear or nonlinear kernel function to map the input space to a higher dimensional feature space and to model and predict the feature space [31]. The model formulation of SVR can be expressed as:

$$\boldsymbol f\left(\boldsymbol x\right)=\beta_0+{\boldsymbol h(\boldsymbol x)}^T\boldsymbol\beta$$

where ${\varvec{f}}\left({\varvec{x}}\right)$ is the vector of breed composition, ${\boldsymbol h(\boldsymbol x)}^{\boldsymbol T}\boldsymbol\beta$ is the kernel function, $\boldsymbol\beta$ is the vector of weights, and ${\beta }_{0}$ is the bias.

### Multi-layer perceptron

**Fig. 2:**
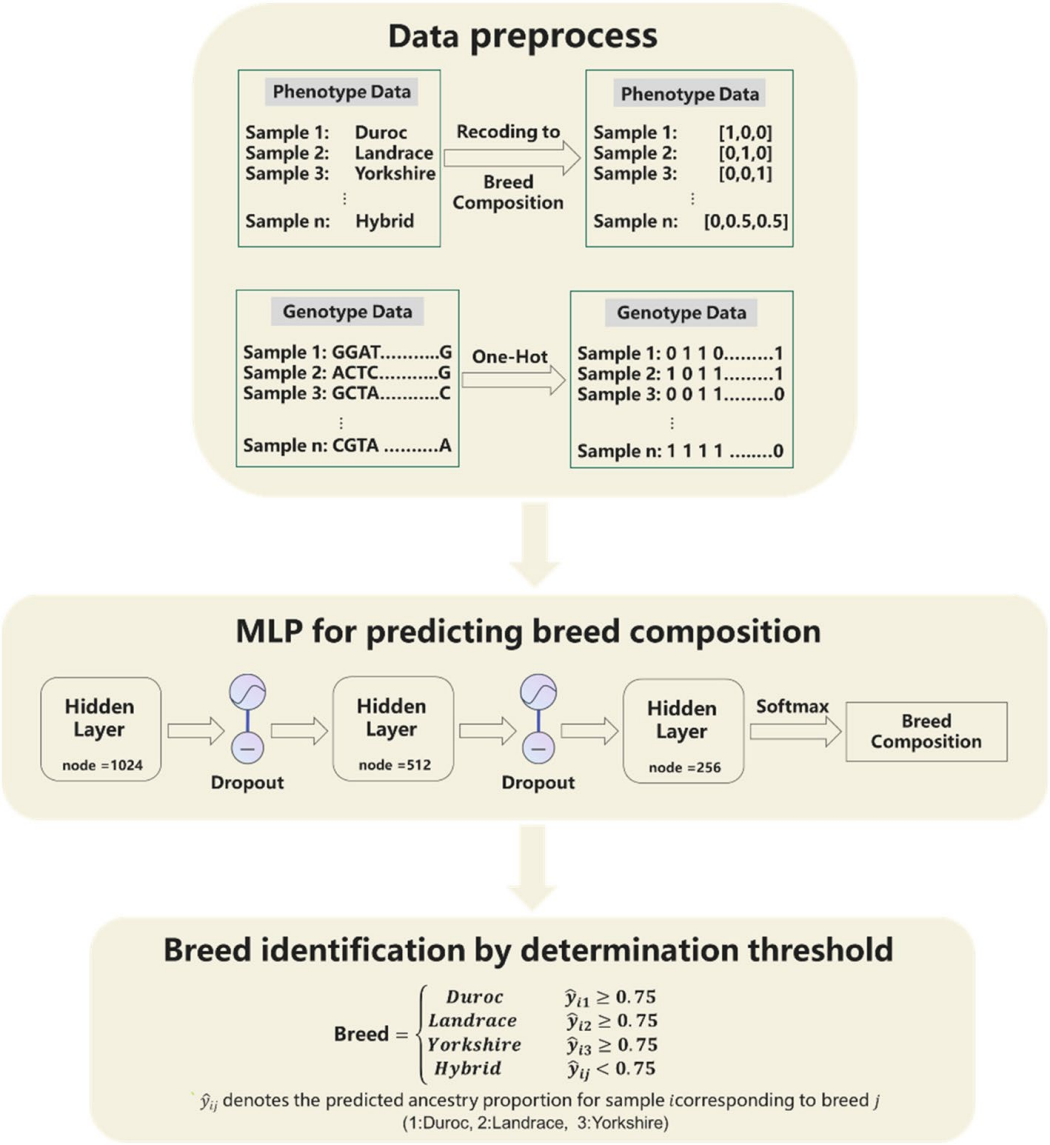
Workflow of the algorithm implementation. Data preprocessing: the genotype data encoded as ATCG is quality-controlled and then re-encoded into binary form using One-Hot encoding; phenotype data related to breeds is re-encoded into breed composition. MLP for predicting breed composition: the re-encoded genotype and phenotype data are fed into an MLP model for training, after which the model predicts the breed composition for individuals in the test set using only their genotype data. Breed identification by determination threshold: breed identification is conducted based on the MLP-predicted breed composition and a determination threshold (0.75)

Figure 2 is the workflow of the algorithm implementation and MLP detailed network structure. MLP is a type of feedforward neural network model consisting of an input layer, one or more hidden layers, and an output layer. Nodes in each layer are connected to the nodes in the next layer through weighted connections, but there are no connections between nodes within the same layer. MLP is capable of learning complex features in the input data by utilizing nonlinear activation functions [32]. It updates weights and biases through backpropagation of errors to minimize the difference between predicted and actual values, thereby effectively optimizing the model parameters. The model formulation of MLP can be expressed as:

$$\boldsymbol y=g({\boldsymbol W}_l(f_{l-1}\left({\boldsymbol W}_{l-1}\left(\dots f_1\left({\boldsymbol W}_1\boldsymbol x+{\boldsymbol b}_{\mathbf1}\right)+{\boldsymbol b}_{\boldsymbol l\boldsymbol-\mathbf1}\right)\dots\right))+{\boldsymbol b}_{\boldsymbol l})$$

where $\boldsymbol{y}$ is the vector of breed composition, ${\boldsymbol W}_l$ is the weight matrix of $l$ layer, ${\boldsymbol b}_l$ is the bias vector of $l$ layer, $f(\cdot )$ is the activation function of $l$ layer, which was set to ReLU in this research, $g(\cdot )$ is the activation function of the output layer, which was set to softmax in this research.

### Model training

Grid search was used to find the optimal hyperparameters for ML methods and a five-fold cross-validation (fivefold CV) strategy was performed to tune the hyperparameters in a grid search. The hyperparameters optimized for SVR encompassed the kernel type (kernel), regularization parameter (C), kernel coefficient (gamma), and polynomial degree (degree). For RF, the hyperparameter search space included the number of estimators (n_estimators), maximum tree depth (max_depth), and maximum features considered during node splitting (max_features). In the MLP, hyperparameter optimization involved learning rate (lr), hidden layer architectures, dropout probability (dropout), and L2 regularization strength (L2). Systematic tuning of these hyperparameters constituted a critical strategy to mitigate model overfitting. Additionally, an early stopping mechanism was integrated into the MLP training protocol to further counteract overfitting by terminating training upon validation loss plateauing. MSE (mean square error) was used to determine the optimal hyperparameters. The hyperparameter grid search results of three ML methods are in Table S1.

### The accuracy of breed identification

Once the optimal hyperparameters for each ML method was assigned, they will be finally used to predict the genomic breed composition of each individual. The threshold value to determine one pure breed was set 0.6, 0.65, 0.70, 0.75, 0.80, 0.85 and 0.90. Two ways were employed to evaluate the breed prediction accuracy of all methods, on one hand, five replicates of fivefold CV were carried out in Population 1, the training and test sets for each repetition were same across all methods and chips. On the other hand, independent testing of using Population 1 to predict Population 2 was implemented for practical identification. In detail, the prediction accuracy was measured as the proportion of correctly predicted breed individuals in the test set.

## Results

### The accuracy of different methods for breed identification

**Fig. 3:**
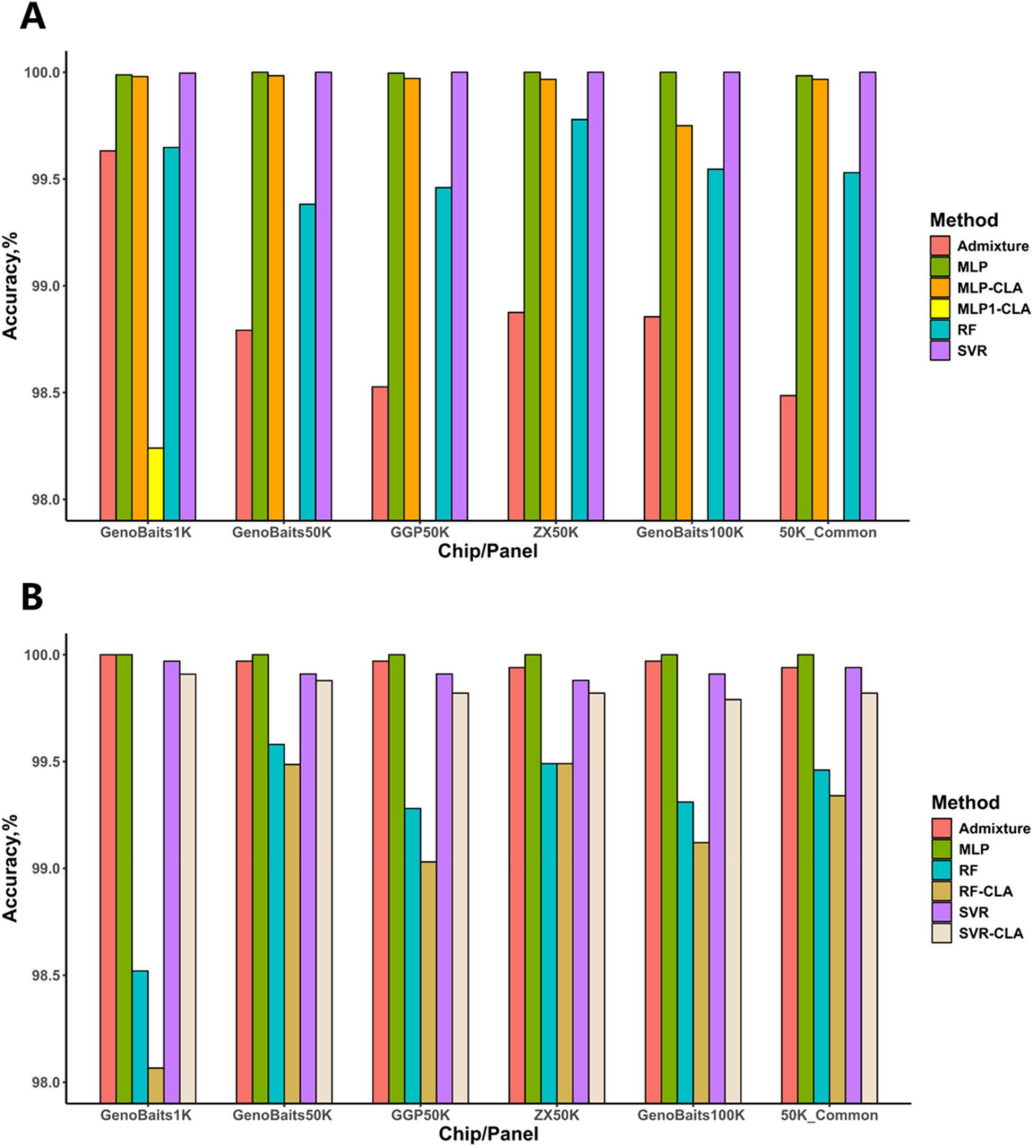
Breed identification accuracy of six methods on six SNP chips/panels in (**A**) five replicates of fivefold cross validation in Population 1, and in (**B**) independent testing of using Population 1 predicting Population 2. Only prediction accuracies greater than 98% are shown. MLP: Multi-Layer Perceptron, RF: Random Forest, SVR: Support Vector Regression, MLP1-CLA: the MLP model architecture with one hidden layer and 30 nodes and classification-based mechanism, MLP-CLA: same model architecture as MLP but with classification-based framework, RF-CLA and SVR-CLA: same model architecture as RF and SVR but both with classification-based framework

Figure 3A and Table S2 present the performance of four breed identification methods at their optimal decision thresholds using fivefold CV in Population 1. The MLP algorithm demonstrated exceptional prediction accuracy across all scenarios. For purebred identification, MLP achieved perfect classification (100% accuracy) for all breeds using 50K SNP chips, GenoBaits100K and 50K_Common. Hybrid identification accuracy reached 100% with GenoBaits50K, ZX50K, and GenoBaits100K, while minor misclassifications occurred with GGP50K (1 error; 99.96% accuracy) and 50K_Common (4 errors; 99.84% accuracy). Notably, even when using the low-density GenoBaits1K panel, MLP maintained 100% accuracy for Yorkshire and Duroc, with near-perfect performance for Landrace (1 error; 99.98%) and hybrid (3 errors; 99.92%). SVR exhibited comparable performance to MLP, achieving 100% accuracy for all pure breeds and hybrid across five SNP panels, with only one hybrid misclassification observed in the GenoBaits1K. RF also performed stably and obtained accuracy above 99.3% on average, while it did not gain accuracy of 100% in all scenarios. In contrast to the ML approaches, Admixture showed variable performance across breed categories. While achieving perfect discrimination for Yorkshire and Duroc, its accuracy substantially decreased for hybrid identification (89.41%), highlighting methodological limitations in hybrid classification.

Besides investigating the performance of all models in Population 1 through cross-validation study, we further validated their robustness using Population 1 to predict the GBC of individuals in Population 2. As Fig. 3B and Table S3 indicated, MLP demonstrated excellent accurate and robustness prediction again, it reached accuracy of 100% for all three pure breeds and hybrid across all SNP chips and panels. SVR experienced a decline in prediction accuracy, yielding 0.026%—0.121% lower accuracy than MLP in same scenario, it did not obtain accuracy of 100% for all purebreds and hybrid as it did in cross validation study. RF showed a slight decrease in prediction accuracy compared to cross-validation study, with a reduction ranging from 0.074% to 1.128%, although the averaged prediction accuracy using the five SNP chips remained above 98.5% and reached highest accuracy of 99.49% with ZX50K. Compared to purebred identification, RF yielded lower accuracy in hybrid identification, it generated the lowest accuracy of 57.84% with 1K SNP chip, and the accuracies for Genobaits50K, ZX50K, GGP50K, Genobaits100K and 50K_common were 93.14%, 87.26%, 85.29%, 83.33%, 91.18%, respectively. They were much lower than those obtained by MLP and SVR. Different from the performance in CV study, Admixture showed a markable improvement in prediction accuracy, it achieved accuracy of 100% for purebreds in all SNP chips/panel, and the accuracies for hybrid was over 98%.

### The advantage of multi-output regression framework for breed identification

In order to further demonstrate the superiority of our improved MLP in this study, we evaluated two classical MLP models (MLP-CLA and MLP1-CLA) for comparison. The MLP1-CLA architecture followed previous methodologies [26, 33], employing a single hidden layer with 30 nodes and a classification-based framework. While MLP-CLA adopted our modified network architecture as in the proposed MLP model but maintained the classification-based framework. As expected, MLP1-CLA exhibited the poorest performance among all evaluated methods (Fig. 3A, Table S2). Although achieving peak average accuracy (98.24%) with the 1K SNP chip, its performance showed an inverse correlation with SNP density accuracy plummeted to 63.45% using the GenoBaits100K panel. Particularly, it should be noted that its hybrid identification capability is unreliable (Table S2). Although the MLP-CLA demonstrated significantly improved accuracy compared to MLP1-CLA, it still performed worse than our proposed MLP.

Table S3 and Fig. 3B further indicated that the multi-output regression framework was more powerful than the classification-based framework. In independent test using Population 1 to predict Population 2, we compared SVR and RF with classification-based framework (designated as SVR-CLA and RF-CLA) to their multi-output regression-based counterparts SVR and RF, respectively. Both SVR-CLA and RF-CLA demonstrated lower prediction accuracy in all cases. The largest performance gap emerged in GGP50K, where RF-CLA generated 32 misclassifications, reflecting a 25% accuracy degradation compared to RF. Particularly, the multi-output regression framework demonstrated advantages in hybrid identification. For RF models, prediction accuracy improved by 1.96–5.89 percentage points across five chips/panel (excluding ZX50K with no improvement). SVR models achieved higher accuracy than SVR-CLA across all six chips/panel, with the most substantial improvement (2.94 percentage points) observed in the 50K_Common panel. Our results confirmed that multi-output regression framework was generally useful for machine learning methods in breed identification, especially in hybrid identification.

### The impact of determination threshold for purebred

**Fig. 4:**
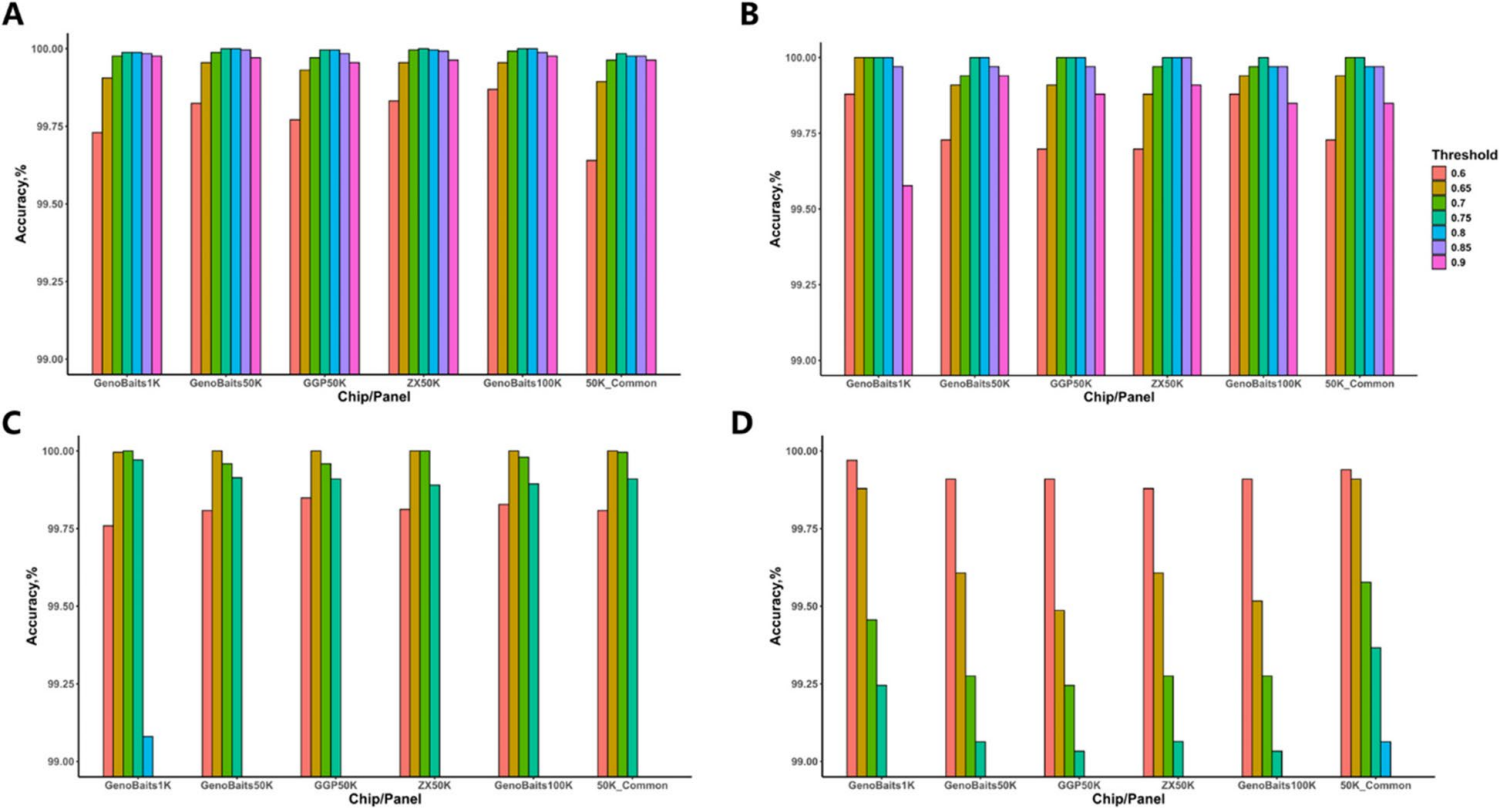
The prediction accuracy of all breeds obtained by MLP and SVR at different determination threshold for purebred. **A** and **B** The performance of the MLP in five replicates of fivefold cross validation in Population 1 and independent testing of using Population 1 predicting Population 2. **C** and **D** The performance of the SVR in cross validation study and independent testing, respectively. Only thresholds with prediction accuracies greater than 99% are shown

The accurate setting of the purebred determination threshold is crucial for breed identification. We investigated the breed identification accuracy of all methods as determination threshold value ranged from 0.6 to 0.9. As shown in Fig. 4 and Table S4, the prediction accuracies of each method varied across different thresholds. MLP had the smallest prediction accuracy variation, it yielded accuracy greater than 99.58% in all situations. Interestingly, across all SNP chips and the 50K_common panel, the prediction accuracy of the MLP first increased and then decreased as the threshold increased, reaching its peak at a threshold of 0.75. Therefore, 0.75 was selected as the optimal threshold for both cross-validation and independent testing (Fig. 3 and Tables S1, S2). SVR and Admixture were sensitive to the thresholds, their accuracies were quite as low as 12.22% and 69.63% in Genobaits50K at high threshold of 0.9, while the accuracies were 62.01% and 84.60% at threshold of 0.85. Their optimal thresholds were different with different SNP chips, and the prediction accuracy fluctuated with thresholds. Both methods reached the highest prediction accuracy at thresholds of 0.60 or 0.65 in most situations. For RF, the variation of prediction accuracy at different thresholds was not as large as SVR and Admixture did. The prediction accuracy was greater than 97% in all situations, it achieved highest accuracy at threshold of 0.7 across all SNP chips and panel for the CV study, while the optimal thresholds varied between 0.6 and 0.75 in independent testing, nevertheless, the difference among these predict accuracies were very tiny.

### The impact of purebred and hybrid individual size in training set

In order to determine the minimum requirement for the number of purebred and hybrid individuals in the training set, the impact of purebred and hybrid individual size in training set on breed identification was investigated. For simplicity, we presented the predict accuracy on Landrace obtained by MLP. As shown in Table 1, in the case of no Landrace individuals in Population 1, MLP was completely unable to correctly identify Landrace. With the number of Landrace individuals in training set increased from 50 to 300, the prediction accuracies with six SNP chips/panel were dramatically increased from 98.28% to 100%. When the number of Landrace individuals increased to 150, the prediction accuracy quickly reached 100%. We further explored the scenario with no hybrid individuals in the training set. As Table 1 indicated, a larger number of Landrace individuals in training set was required to acquire the comparable accuracy in the scenario with 491 hybrid individuals, implying hybrid was helpful to improve the predict accuracy of purebreds. Moreover, the SNP density only took slight effect on the accuracy. The effect of hybrid individual size in training set on hybrid identification accuracy was also similar to that of the Landrace study (Table S5).

**Table 1:** The impact of number of Landrace individuals in training set on the Landrace identification accuracy

The number of individuals in training set				Landrace identification accuracy					
Yorkshire	Duroc	Hybrid	Landrace	GenoBaits1K, %	GGP50K, %	ZX50K, %	GenoBaits50K, %	GenoBaits100K, %	50K_Common, %
2,185	1,111	491	0	0	0.54	1.27	0	2.17	0.18
			50	99.09	99.09	98.37	99.28	98.55	98.55
			100	99.82	99.82	99.64	99.82	99.28	99.46
			150	100	100	99.64	99.82	99.82	100
			200	100	100	99.64	99.82	100	100
			250	100	100	99.64	99.82	100	100
			300	100	100	100	100	100	100
		0	0	0	0	0	0	0	0
			50	99.46	99.09	99.09	99.09	98.19	98.19
			100	99.64	99.64	99.28	99.09	99.10	99.46
			150	99.82	99.64	99.28	99.46	99.09	99.64
			200	99.82	99.82	99.46	99.64	99.09	99.82
			250	99.82	99.82	99.82	99.82	99.82	99.82
			300	99.82	99.82	99.82	100	100	99.82
			350	100	99.82	100	100	100	99.82
			400	100	99.82	100	100	100	100

The Landrace in the test set were from Population 2

### The impact of SNP density

Despite the significant differences in density among the five SNP chips, our results did not show a consistent trend of higher density correlated with higher prediction accuracy. In fact, Admixture with the lowest-density chip of GenoBaits1K exhibited the highest prediction accuracy in Population 1 (Fig. 3A and Table S2). For MLP, all five SNP chips achieved the highest prediction accuracy, demonstrating that using these five chips in conjunction with MLP for breed identification is optimal. Although GenoBaits1K had slightly lower prediction accuracy in some cases, it still remained above 99.99%. In addition, utilizing the intersecting SNPs from the three 50K chips (50K_Common) for breed identification would be useful. The performance of 50K_Common across the four methods was comparable to the three 50K SNP chips, and it achieved the highest prediction accuracy obtained by MLP (Fig. 4 and Tables S2, S3).

## Discussion

Currently, researches on breed identification in livestock mainly focused on purebreds, and the accuracy of such identifications has yet to reach 100%. Only a few studies partially covered the hybrid identification, and they yielded the lower predict accuracy in hybrid than in pure breeds [26, 34], which could not meet the requirements from practical pig breeding. In this study, we proposed a novel breeding identification strategy that initially employs an enhanced MLP architecture, specifically developed in this study, for GBC prediction. Subsequently, the breed is determined through a predefined threshold criterion. Our enhanced MLP architecture incorporates two synergistic innovations: 1) structural optimization through a deepened hierarchical network (1024-512-256 node configuration across three hidden layers), and 2) employing a multi-output regression framework supersedes conventional categorical classification framework, further improving the predict accuracy. The poorer performance of MLP1-CLA as investigated in previous studies [26, 33]demonstrated the significant improvement of our proposed MLP algorithms with multiple hidden layers. Our results also confirmed the superiority of multi-output regression framework to the classification-based framework (MLP-CLA), particularly in hybrid identification (Fig. 3A, Table S2). Traditional classification-based frameworks [24, 25, 34] face two fundamental limitations. Firstly, they risk misclassifying hybrids with high ancestral purity (> 50% single-breed contribution) as purebreds due to threshold-based decision boundaries. Secondly, class imbalance in training data induces prediction bias toward overrepresented breeds [35]. Our multi-output regression framework effectively circumvents these issues by generating continuous GBC estimates rather than discrete classifications. This contrasts with classification models employing cross-entropy loss functions, which prioritize maximum likelihood estimation of categorical labels rather than precise ancestral quantification [36].

Our results demonstrated that MLP model with multiple layers was highly effective, it generally outperformed other two ML methods and Admixture, achieving a breed identification accuracy of 100% for both hybrid and purebreds in 50K and 100K SNP chips, e.g. MLP completely accurately identified three pure breeds (Duroc, Landrace and Yorkshire) and crossbred of Landrace with Yorkshire using two 50K SNP chips (GenoBaits50K and ZX50K) in both cross validation study and independent testing (Fig. 3, Tables S2 and S3). On the other hand, the implementation of multi-out regression framework in MLP, RF and SVR architectures yielded substantial performance improvements over classification-based framework used in previous studies (Fig. 3, Tables S2 and S3). Our results demonstrated that the multi-output regression framework achieved consistent improvements over classification-based framework across all six SNP panels. The multi-output regression framework exhibited pronounced advantages in hybrid identification, with accuracy improvements reaching up to 5.89 percentage points than classification-based framework. In addition, our optimized RF (99.78% accuracy) and SVR (99.91% accuracy) models surpassed the 92.72%–97.56% range reported by Miao et al. [34] and the 98.11%–99.24% accuracy achieved through feature-selected SNP panels in Zhao et al. [33].

In this study, we compared our method with two ML methods RF and SVR as they frequently employed high accuracy in variety identification research [11, 26, 33, 34]. In addition, XGBoost and Transformer models are currently the most prominent methodologies, while rare researches on breed identification are available by far. We hereby only evaluated the performance of XGBoost in independent testing of using Population 1 to predict Population 2 (Table S6), the prediction accuracy achieved by XGBoost was substantially lower than that of SVR, RF and MLP. Hornstein et al. [37] pointed out Transformer contain an excessive number of parameters and demand substantial sample sizes, approximately 50,000 samples are required to achieve high model performance. Our results showed that, under the multi-output regression framework, MLP yielded higher prediction accuracy than RF and SVR. It is inconsistent with the findings reported by Xu et al. [26] and Zhao et al. [33], in which SVM and RF methods outperformed MLP. It might be due to the relative simple structure of MLP model in their studies, only one hidden layer with 30 nodes made it incapable of learning high-dimensional genomic patterns, thereby limiting the performance of the MLP [32, 38]. It was confirmed by our results from MLP with one hidden layer (MLP1-CLA in Fig. 3A and Table S2), in which MLP1-CLA performed worst among all methods and exhibited pronounced sensitivity to SNP chip density, rendering it unsuitable for medium-to-high density genomic applications. By contrast, our optimized three-layer architecture (1024-512-256 nodes) enabled effective learning of complex feature interactions, achieving significant accuracy improvements.

The superior performance of MLP over RF and SVR is reasonable. MLP can expand its model capacity by increasing the number of layers and nodes, allowing it to fit more complex functions better [32]. In contrast, RF and SVR typically have fixed model capacities, and although their complexity can be altered by adjusting parameters, they do not offer the same performance as MLP in terms of model capacity [38]. When all the SNPs on the chip were used as input, redundancy and noise were usually generated. MLP autonomously performs hierarchical representation learning through successive nonlinear transformations, effectively distilling discriminative breed-specific signatures from high-dimensional inputs [39]. However, RF and SVR cannot perform representation learning on their own and rely heavily on the quality of the input features. Hermes et al. [40] found that the generalization ability of the SVM improved after the stepwise removal of noise-contaminated features. Kigo et al. [41] also observed in RF applications that failure to process redundant features leads to overfitting. Our study further confirmed this perspective, as SVR and RF exhibited slight adaptation to localized noise specific to Population 1, resulting in comparatively reduced predictive accuracy on Population 2.

Different from other studies [11, 42–44], our strategy involves outputting the GBC for each individual and then applying breed-specific thresholds for identification, which aligns better with genetic principles. We set seven different threshold levels from 0.6 to 0.90, and our findings showed the determination threshold value is also a key factor influencing prediction accuracy. It could be seen that even a slight change of 0.05 in the threshold value took a significant effect. For instance, when using SVR with the GenoBaits50K chip for breed identification, increasing the determination threshold from 0.8 to 0.85, dramatically reduced the prediction accuracy from 98.00% to 62.01%. Among all methods, MLP was the most suitable method for breed identification using threshold value determination, as it not only achieved high accuracy but also demonstrated robustness of the determination threshold value across populations. Its optimal threshold was 0.75 as the results of the cross validation and independent testing cross all SNP chips/panel indicated (Fig. 3 and Tables S2–S4). Although the threshold was very close to the hybrid (50% admixture), the misclassification of hybrid as purebred was very low. For the breed with the highest proportion in the GBC predicted by MLP for all hybrid individuals of the six chips/panel, the proportion of the most prevalent breed's genetic lineage (Landrace or Yorkshire) was generally between 0.45 and 0.65 among 99.10% to 99.45% hybrid individuals, and the proportion of Duroc ancestry was less than 0.01 across all the samples (Table S7), which was consistent with the biological characteristics of the hybrid.

SVR, Admixture and RF were very sensitive to threshold values, their predict accuracy fluctuated with thresholds in cross validation and independent testing in different SNP chips, e.g. SVR yielding optimal threshold of 0.65 in CV study and 0.60 in independent testing. Because we carried out 5 replicates of CV study and the variations of predict accuracy within CV and between replicates were very tiny, the results were more stable. Their optimal threshold values were 0.70, 0.65 and 0.65 for SVR, RF and Admixture, respectively, which were close to 0.75 obtained by MLP. Our results suggested that 0.65–0.75 is ideal threshold valued for accurate breed identification. This is differing from the conventional practice of using a manually determined threshold of 0.9 to distinguish between purebreds and hybrids [9]. Miao et al. [34] has addressed threshold determination, however, they did not account for the ancestry patterns of hybrids, classifying an individual as a hybrid if all purebred label probabilities were below 0.03, leading to lower accuracy for hybrid identification.

The training set size is crucial for breed identification. Miao et al. [34] used a reference panel consisting of 2,272 individuals and 45,743 SNPs and comprised 91 breeds, of which 41 were Chinese indigenous breeds, which yielded 98.4% accuracy on average using ML methods. Wilmot et al. [24] reported a highest accuracy of 98.33% in breed identification using a cohort of 562 cattle from 12 breeds, even when training the model with a carefully selected set of 7,153 SNPs. In the independent testing in this study, the reference population size was 4,889, it was large enough to generate the 100% accuracy for MLP and over 99.3% accuracy for other ML models and Admixture. Even using 1K SNP chip (GenoBaits1K), the accuracy obtained by ML and SVR was 100%, Admixture and RF was 99.7% and 98.52%. Our results also showed the composition of training set is important, the absence of a particular breed in the training set made it impossible to correctly identify this breed. The findings was confirmed by Xu et al. [26] in which prediction accuracy reached 99.30% when the training set includes all breeds present in the test set, however, for the test breeds not represented in the training set, prediction accuracy dropped to 85.68%. Similar conclusions were also drawn by Miao et al. [34].

In addition, the hybrid individuals in the training set is helpful to improve the predict accuracy either for purebred or for hybrid (Tables 1 and S5), because hybrid is derived from the crossbreeding of Landrace and Yorkshire and share genetic similarities, they exhibit unique phenotypic traits and higher genetic diversity [45, 46]. These make it challenging to extract relevant features solely from a purebred training set. As the number of hybrid individuals in training set increased from 0 to 300, almost all chip densities achieved 100% prediction accuracy, with some chips reaching 100% accuracy at 150 individuals. A larger number of individuals in our study particularly for MLP is needed, as greater number of model parameters require more samples to fit the model [33, 43]. In this study, 8,199 pigs sourced from breeding farms in eight provinces in China, could be an ideal training population for the breed identification in pigs. It is more representative and larger than those in previous studies, which can guarantee the optimal model training and predict the Yorkshire, Landrace, Duroc, and crossbred of Yorkshire × Landrace with different genetic background, as the population in this study covering different genetic background.

In this study, we investigated the efficiency of several different density SNP chips, which were popular for pig genotyping. Generally speaking, all methods yielded highest predict accuracy using 50K and 100K SNP chips, particularly, MLP gained 100% accuracy using GenoBaits50K and ZX50K in CV study and independent testing (Fig. 3, Tables S2 and S3). Meanwhile, the 1K SNP chip (GenoBaits1K) could also obtain comparable high accuracy with 50K and 100K SNP chips in most situations. For instance, MLP and SVR yielded 99.988% and 99.996% accuracy on average in cross validation study, and 100% and 99.97% accuracy in independent testing (Tables S2 and S3). GenoBaits1K will become attractive as it is significantly less expensive, leading to a substantial reduction in cost [47]. On the other hand, as more and more company has implemented genomic selection in practical breeding, in which 50K SNP is widely used, breeding identification could be byproduct. For the company already possessed data from more than one kind of 50K chips, breed identification can be performed using the common SNPs from different 50K chips as our results show, allowing for the maximum utilization of existing data to build the training set and reduce the cost of constructing the training set.

## Conclusions

In this study, we proposed a novel multi-output regression MLP architecture for breed identification in pigs. The proposed MLP performed best in the comparison with RF, SVR and Admixture, it was applicable across low-, medium-, and high-density SNP chips. Multi-output regression framework is also helpful to improve the accuracy for RF and SVR, yielding higher accuracy than classification-based framework. The determination threshold of 0.65–0.75 for purebred is ideal threshold value for accurate breed identification for all methods. Among different density SNP chips, low density of 1K SNP chip is more cost-effective as yielding 100% accuracy with increasing training set. Our method is also useful for breed identification in other livestock.

## Supplementary Information

Supplementary Material 1: Table S1 Hyperparameter grid search results. Table S2 Breed identification accuracies in five replicates of fivefold cross validation study in Population 1. Table S3 Breed identification accuracies in independent testing of using Population 1 to predict Population 2. Table S4 The prediction accuracies of all breeds at different determination threshold values for purebred. Table S5 The identification accuracies of hybrid at different number of hybrid individuals in training set. Table S6 Breed identification accuracies in independent testing of using Population 1 to predict Population 2 for XGBoost. Table S7 Distribution of the proportion of the breed with the highest proportion in the genomic breed composition of all hybrid individuals predicted by MLP.

## Data Availability

The datasets used or analyzed during the present study are available from the corresponding author on reasonable request.

## Abbreviations

50K_Common: 50K common SNP panel
fivefold CV: Five-fold cross-validation
AI: Artificial intelligence
ANN: Artificial neural network
GBC: Genomic breed composition
GenoBaits100K: Molbreeding GenoBaits^®^ Porcine 100K
GenoBaits1K: Molbreeding GenoBaits^®^ Porcine DK 1K
GenoBaits50K: Molbreeding GenoBaits^®^ Porcine DK 50K
GGP50K: Illumina Porcine GGP 50K
ML: Machine learning
MLP: Multi-layer perceptron
MSE: Mean squared error
PLS-DA: Partial least squares discriminant analysis
RF: Random forest
SVM: Support vector machines
SVR: Support vector regression
ZX50K: Zhongxin No.1 50K
